# Natural products against inflammation and atherosclerosis: Targeting on gut microbiota

**DOI:** 10.3389/fmicb.2022.997056

**Published:** 2022-12-01

**Authors:** Bing Deng, Liyu Tao, Yiru Wang

**Affiliations:** ^1^Longhua Hospital, Shanghai University of Traditional Chinese Medicine, Shanghai, China; ^2^Shuguang Hospital, Shanghai University of Traditional Chinese Medicine, Shanghai, China

**Keywords:** gut microbiota, atherosclerosis, inflammation, natural products, immune

## Abstract

The gut microbiota (GM) has become recognized as a crucial element in preserving human fitness and influencing disease consequences. Commensal and pathogenic gut microorganisms are correlated with pathological progress in atherosclerosis (AS). GM may thus be a promising therapeutic target for AS. Natural products with cardioprotective qualities might improve the inflammation of AS by modulating the GM ecosystem, opening new avenues for researches and therapies. However, it is unclear what components of natural products are useful and what the actual mechanisms are. In this review, we have summarized the natural products relieving inflammation of AS by regulating the GM balance and active metabolites produced by GM.

## Introduction

More than 3,000 distinct bacterial species, or trillions of microbes, can be found in the human gut, with each bacterium housing 200–300 different species (Qin et al., 2010). This microbial ecosystem is also known as the gut microbiota (GM). However, the specific taxa and the functional composition of our microbiome cannot be detected by culture-dependent bacterial identification techniques. We now have innovative methods to investigate these variations, including biological and enzymatic properties of our microbiome by whole genome shotgun sequencing (Koedooder et al., 2019).

Some evidence suggests that GM play roles in inflammation, nutrient absorption, vitamin production and xenobiotic metabolism (Milani et al., 2017). Despite our long-standing relationship with microbes, we are now beginning to realize their symbiotic dependence, especially with diseases caused by the imbalance between harmful and beneficial bacteria (Zhang and Chu, 2021). The GM has now been well characterized in models of inflammatory and autoimmune disease (Clemente et al., 2018). Research suggests that GM are critical environmental factors in the regulation of atherosclerosis (AS) inflammation (Vourakis et al., 2021). When there is an incidence of disease or infection, the mucosal immune system must have the ability to selectively and actively tolerate the GM under steady-state conditions while being able to mount an appropriate inflammatory response.

Natural products are the foundation of modern medicine and have been used to develop numerous pharmaceutical medications. Morphine (Thompson et al., 2022), strychnine (Zlotos et al., 2022), ephedrine (Yu et al., 2018), paclitaxel (Zhu and Chen, 2019), artemisinin, which earned the 2015 Nobel Prize in Physiology or Medicine, are among the most well-known. Because of their benefits to human health, natural products have been proved to be important in current medical research and innovation. Only a few active components of natural products have been found by *in vitro* and *in vivo* pharmacological and physiological evaluation. Natural medicines have been the focus of much research over the last decade, and their usage as supplementary and alternative treatments is already on the sharp rise.

Natural products are well-known for containing a large number of different components and being extremely bioactive. These substances are used to create new pharmacological treatments to treat disorders, such as cardiovascular disease (CVD) and AS through regulating GM (Zhou and Wang, 2014; Zhao et al., 2021). More research and development are needed to completely comprehend the core mechanism of action. Insights about how natural products might alter local and systemic inflammation in the human GM-AS disease axis will be discussed in this review.

## Burden of cardiovascular disease

CVD (which includes coronary heart disease, heart failure, stroke, and hypertension) is becoming more prevalent among both males and females under the age of 20 (126.9 million in 2018). It is the leading cause of mortality worldwide, accounting for 18.6 million deaths (95% confidence interval: 17.1–19.7 million) in 2019, an increase of 17.1% (95% confidence interval: 11.4–22.9%) from 2010. In addition to the increased burden of morbidity and mortality, CVD currently represents the biggest direct cost ($96.2 billion) in 2016–2017. As a result, CVD is significantly linked to a higher death rate, a greater use of healthcare resources, and a greater financial burden (Virani et al., 2021).

## Role of inflammation in AS

AS is a chronic degenerative pathological change of large and medium-sized arteries defined by lipid buildup in the arterial wall, accompanied by local neovascularization and apoptosis from the onset to the manifestation of problems (Moroni et al., 2019). AS is not only a major cause of CVD, but also leads to various types of cerebrovascular illness with a high death rate (Herrington et al., 2016). It has surpassed communicable illnesses to become the world’s leading and fastest-growing cause of death and disability (Roth et al., 2020).

### Basic researches

**Figure 1:**
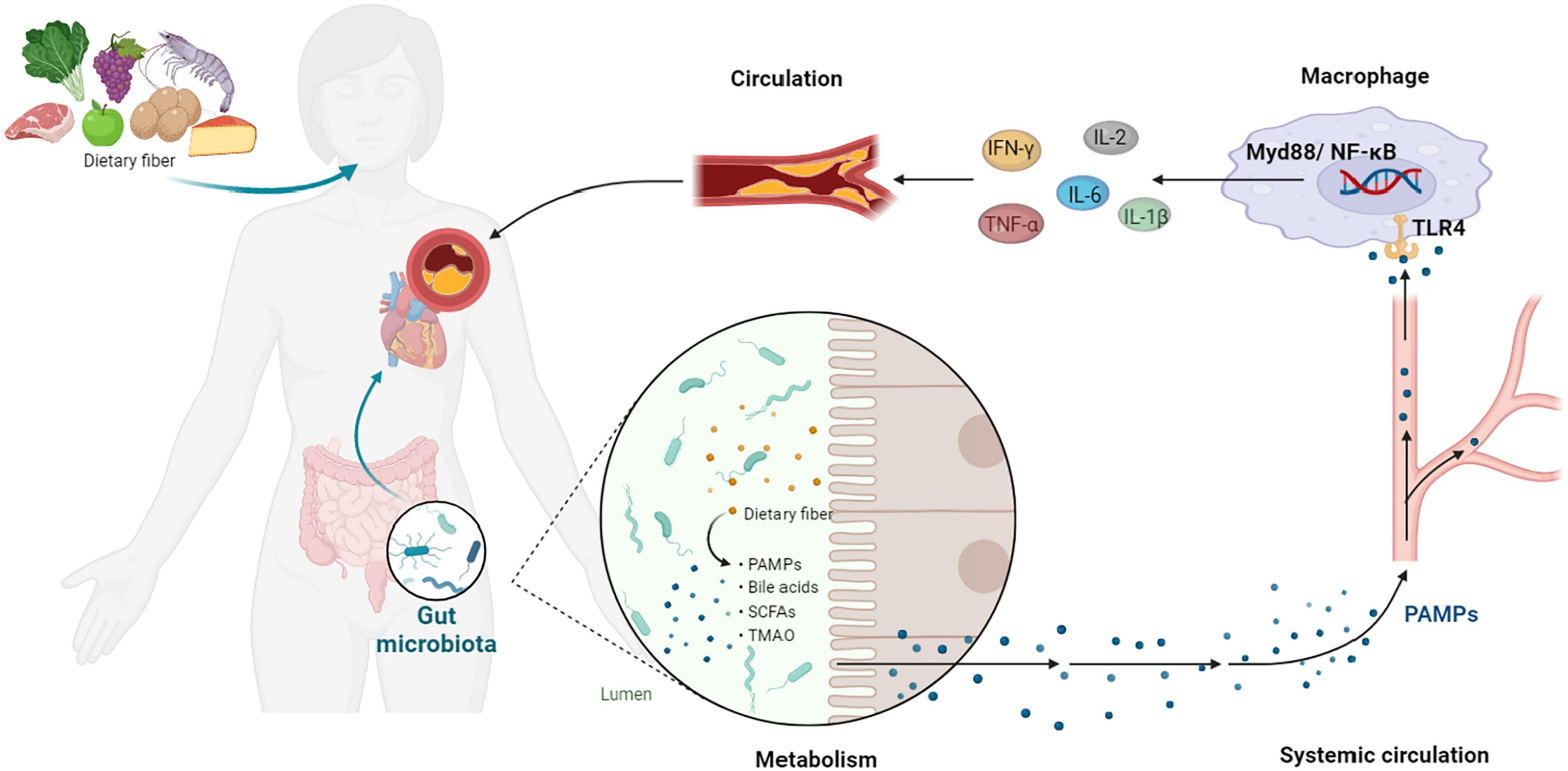
GM-Inflammation axis in AS. PAMPs, pathogen-associated patterns; TLR4, Toll-like receptor 4; NF-κB, NF-kappa B; TNF-α, tumor necrosis factor-α; IL, interleukin; IFN-γ, interferon-γ.

**Figure 2:**
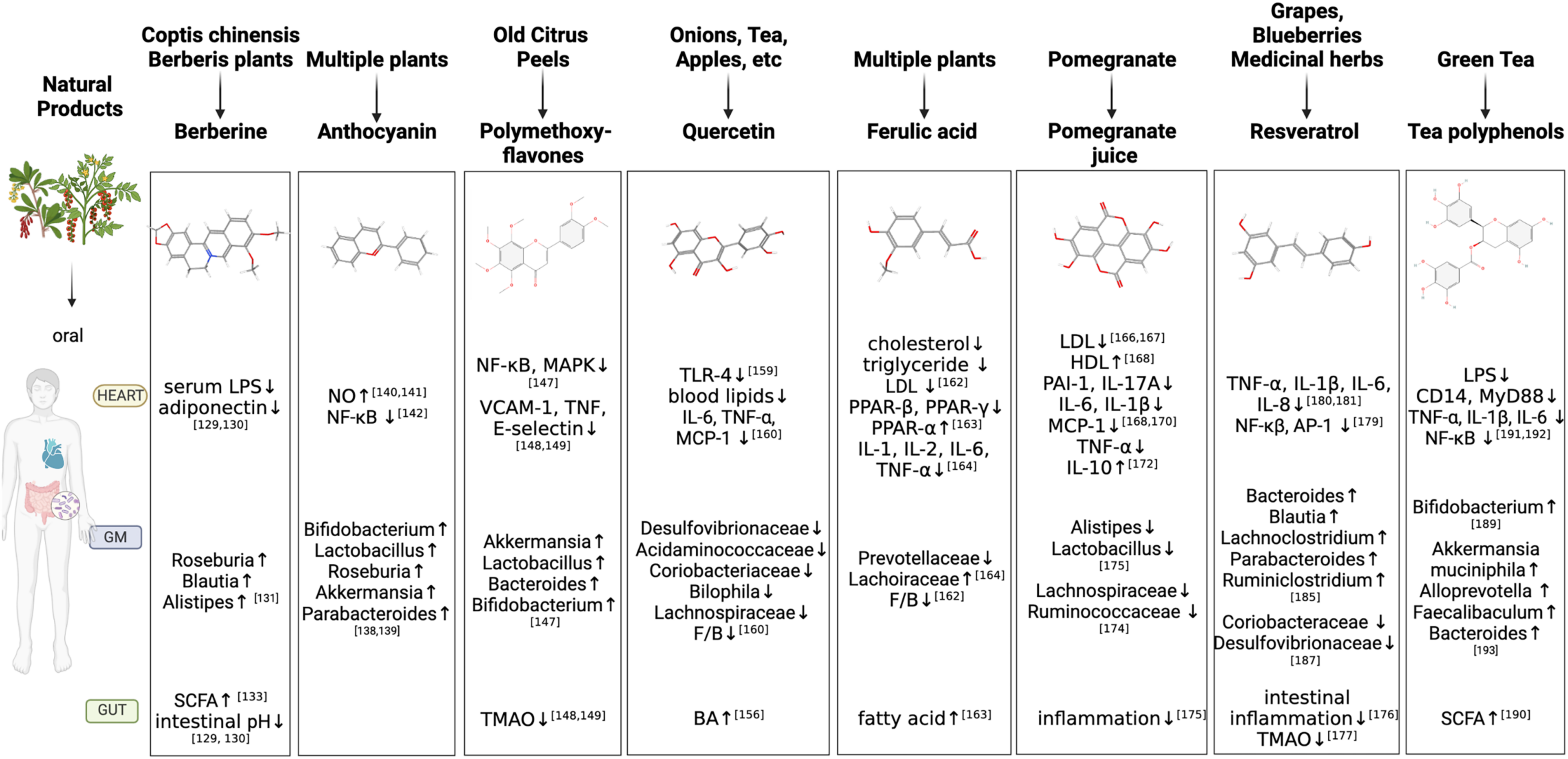
Natural Products regulate GM-inflammation axis in AS. GM, gut microbiota; LPS, Lipopolysaccharide; SCFA, Short-Chain Fatty Acids; NO, nitric oxide; VCAMs, vascular cell adhesion molecules; TNF, tumor necrosis factor; NF-κB, NF-kappa B; MAPK, mitogen-activated protein kinase; TMAO, Trimethylamine N-oxide; TLRs, Toll-like receptors; IL, interleukin; MCP, monocyte chemotactic protein; F/B, Firmicutes/Bacteroidetes; BA, Bile acids; LDL, Low density lipoprotein; PPAR, peroxisome proliferators-activated receptor; IL, interleukin; HDL, high density lipoprotein; PAI-1, plasminogen activator inhibitor-1; AP-1, activator protein-1; ↑, increase; ↓, decrease.

The major pathogenic component that contributes to AS is lipid deposition. Oxidized low-density lipoprotein (ox-LDL) may carry endogenous cholesterol and modulate cholesterol expression in surrounding tissues (Groenen et al., 2021). Macrophages, which are the body’s main phagocytes, may absorb and breakdown Low density lipoprotein (LDL) and ox-LDL (Khoury et al., 2021). When macrophages phagocytose too much lipid and reach their maximal dynamic equilibrium, a mass of macrophage-derived foam cells gather in the injured arteries (Feng et al., 2021). Although AS starts in cholesterol plaques in the artery’s intima, it causes both local and systemic inflammation of both the adaptive and innate immune systems (Montarello et al., 2022). Chronic inflammatory response is thought to occur in three stages of the AS pathological process: early (lipid streak stage), advanced (fibrous plaque, atheromatous plaque formation), and late (fibrous plaque, atheromatous plaque formation; unstable plaque, plaque rupture, and thrombosis) stage (Jing et al., 2022). Ox-LDL and cytokines act on endothelial cells in the early stages of AS, causing them to release vascular cell adhesion molecules (VCAMs), selectin, and other adhesion molecules. Monocytes-macrophages, vascular smooth muscle cells (VSMCs), neutrophil cells, T lymphocytes, and B lymphocytes are among the cytokines and immune cells involved. These cells and cytokines engage with receptors on the cell membrane’s surface, which subsequently activate a number of linked signaling pathways through transmembrane signaling, inducing the production of target genes (Moriya, 2019; Roy et al., 2022). As a result, it’s critical to investigate the signaling route linked to inflammation and develop an effective therapeutic plan based on this target (Figures 1, 2).

### Clinical studies

Inflammation is largely to blame for the higher risk of CVD patients, according to decades of preclinical research. The CANTOS (Canakinumab Anti-inflammatory Thrombosis Outcome Study), which is reported in 2017, is the most important of the IL (interleukin)-1β trials. IL-1β has been linked to atherogenesis in experimental studies for decades. CANTOS find that inhibiting IL-1β decrease recurrent significant adverse cardiovascular events in CAD patients with well-controlled LDL levels, irrespective of cholesterol reduction (Ridker et al., 2017). This groundbreaking study shows that inhibiting the IL-1β/ IL-6 signaling cascade can reduce cardiovascular risk without reducing cholesterol levels, but at the cost of an increased risk of severe illness. CANTOS took inflammatory therapy in AS from a scientific hypothesis to a practical reality.

Following the success of CANTOS, many techniques for interfering with IL-1β or its production have evolved, including inflammasome inhibition. Further to that, the Colchicine Cardiovascular Outcomes Trial (COLCOT) and Low-Dose Colchicine 2 trial (LoDoCo2) demonstrate that treatment with colchicine, an anti-inflammation medication that targets multiple inflammatory pathways, reduces cardiac events in patients with atherosclerotic CVD (Tardif et al., 2019; Nidorf et al., 2020). In patients with recent or momentarily remote acute coronary syndromes (ACS), all of these investigations have shown therapeutic benefit.

Meanwhile, some potential research areas are now under active development but have not yet entered the clinical trial phase. Tocilizumab, a humanized monoclonal antibody targeting the IL-6 receptor, has already been evaluated in patients with ACS. The tocilizumab group has a higher myocardial salvage index than the placebo group, while the placebo group has a lower C-reactive protein (CRP; Kleveland et al., 2016; Broch et al., 2021). In the Cardiovascular Inflammation Reduction trial (CIRT), 4,786 individuals with past ACS and either type 2 diabetes mellitus or metabolic syndrome are randomized to low-dose methotrexate 15 to 20 mg weekly or placebo. Participants in the methotrexate group have lower high sensitivity CRP levels to begin with, and methotrexate have no effect on decreasing IL-1β, IL-6, or high sensitivity CRP levels (Ridker et al., 2019).

As a result, targeting inflammatory pathways might be a potential new method to prevent and cure AS (Kong et al., 2022). Inhibition of the immune response weakens the host’s defenses, making patients more vulnerable to infection. Hence, reducing chronic inflammation in atherosclerotic CVD is a goal. Finding a strategy to do so without affecting the immune system is critical.

## Interplay of GM and AS

Several studies in recent years have verified the presence of bacterial deoxyribonucleic acid (DNA) in atherosclerotic plaques, and the amount of DNA linked with the presence of many leukocytes in the plaque, which may contribute to the CVD development (Koren et al., 2011; Torres et al., 2015). Furthermore, researchers discover that AS patients have abnormalities in their GM when compared to persons without AS (Koren et al., 2011; Baragetti et al., 2021; Ji et al., 2021; Szabo et al., 2021). The impact of GM on the development of atherosclerotic lesions is investigated in a few experiments. Endothelium plaque lesions are reduced in specific-pathogen-free (SPF) model animals compared with germ-free (GF) model animals at various time intervals. At 52 weeks, the SPF group has mild aortic atherosclerotic lesions compared to the GF group, whereas the GF group had significant coronary AS (Liu et al., 2018; Chuang et al., 2022). GM dose not indicate a major role to the late aortic AS of LDL receptor-deficient (LDLR^−/−^) mice in GF environment (Kiouptsi et al., 2020). Chen et al. demonstrate the possibility of altering the GM to prevent the AS onset and progression in LDLR^−/−^ mice, indicating that the GM plays a role in AS and may also give a novel treatment option (Chen et al., 2020).

A higher Firmicutes/Bacteroidetes (F/B) ratio, which is frequently observed in obese persons and can validate the AS preventive effect, is detected in CAD patients according to two clinical investigations (Cui et al., 2017; Szabo et al., 2021). Phylum Proteobacteria, *genus Escherichia* is abundant in individuals with subclinical carotid AS and CAD, providing a novel predictor in the AS progression (Zhu et al., 2018; Baragetti et al., 2021). Phylum Bacillota, *genus Acidaminococcus* is formerly often found in people with inflammatory disorders and a pro-inflammatory diet, also more prevalent in the AS patients (Ji et al., 2021). Mitra et al. (2015) observed that the GM in patients with unstable plaque have more abundant *Helicobacteraceae* and *Neisseriaceae* than individuals with stable plaques. However, Lindskog Jonsson et al. (2017) point that there are no significant variations in bacterial DNA quantity or microbial composition between asymptomatic and symptomatic individuals’ plaques, or between various plaque areas. It indicates that other variables are more crucial in deciding plaque susceptibility.

Finally, there is no complete consensus on whether the GM plays a role in AS development. The relationship between the GM and AS has to be investigated further.

### Altered composition and development of GM

The GM is a collection of microorganisms such as viruses, archaea, fungus, and bacteria that make up the gut’s most prevalent components. The microbiota is most commonly found in the gastrointestinal tract, particularly in the ascending colon, which is largely anaerobic and has a nutrient-rich environment that is conducive to microbe proliferation (Shanahan et al., 2021). The infant’s GM appears to be unstable and lacking in variety, as indicated by the technique of delivery. According to Watson et al., a cesarean section is used to deliver 64–82% of babies infected with methicillin-resistant Phylum Staphylococcus, *genus Staphylococcus aureus* (James et al., 2008). Babies born through cesarean section may be more vulnerable to pathogen infections. In addition, analogous to human skin, the GM of a baby delivered *via* cesarean section is changed and dominated by Phylum Streptococcus, Phylum Corynebacterium, and Phylum Propionibacterium (Rautava, 2017). The intestinal microbiota evolves until around the age of three, when it becomes a varied, complicated, and steady collection with 60–70% resemblance to the adult GM, influenced by the environment surrounding the baby (Kashtanova et al., 2016). Some elements can impact the microbiota throughout development, such as food (i.e., breast milk or formula feeding) and antibiotic usage, which is frequently linked to disturbance of the newborn GM. Several studies have discovered that the GM of infants can promote long-term health and that human GM at an early age is linked to certain adult health issues (Milani et al., 2017; Butel et al., 2018; Kim et al., 2019).

Nine phyla that make up the intestinal flora of adults, which are Actinobacteria, Bacteroidetes, Firmicutes, Fusobacteria, Proteobacteria, Cerrucomicrobia, Lentisphaerae, Verrucomicrobia, Euryarchaeota, and Spirochaetes (Li et al., 2014). Bacteroidetes and Firmicutes make up over 90% of the total bacterial species in a healthy bacterial ecosystem (Qin et al., 2010). Simultaneously, the F/B ratio varies greatly, which is used as a health indicator of the GM in all persons. The F/B ratio rises in obese persons (the phylum Firmicutes rises, while the phylum Bacteroidetes falls) and is linked to several CVD (Crovesy et al., 2020; Magne et al., 2020). It has been demonstrated that the microbial makeup of CAD patients’ changes, with a large rise in Firmicutes abundance and a reduction in Bacteroidetes abundance (Emoto et al., 2016; Sanchez-Rodriguez et al., 2020). The GM evolves not just in the early stages of life, but even in the elderly, with changes in bacterial diversity and shifting of dominating species (e.g., decreasing abundance of beneficial microorganisms and increasing abundance of facultative anaerobic bacteria). These alterations might be linked to the onset of AS in the elderly (Shen et al., 2021; Vourakis et al., 2021). Details are shown in Table 1.

**Table 1:** Gut microbial-derived metabolites and CVD.

Metabolite	Experimental models	Main outcomes	References
TMAO	908 European participants who subsequently develop CVD	A dose-dependent increases in CVD risk with an increase in circulating TMAO levels	Zheng et al. (2019)
	86 newly diagnosed CVD patients	New CVD cases have a higher baseline levels of TMAO	Iglesias-Carres et al. (2021)
	C57BL/6 J and ApoE−/− mice with atherosclerosis	TMAO relates to AS, independent of cholesterol homeostasis	Lindskog Jonsson et al. (2018)
	ApoE−/− mice	Increased plasma TMAO is associated with plasma cholesterol levels	Collot-Teixeira et al. (2007)
	C57BL/6 and BALB/C mice	TMAO can activate NLRP3, which can lead to IL-1β, IL-6 and TNF-α production	Seldin et al. (2016)
	LDLR−/− mice	Acute TMAO injection induces inflammatory markers and activates the MAPK, ERK and NF- κB signaling cascade	Chou et al. (2019)
	81 stable angina patients	Plasma TMAO levels are negatively correlated with circulating EPC numbers and the FMD, and positively correlates with hsCRP, IL-1β concentrations	Chen et al. (2017)
	ApoE−/− mice	TMAO promotes NLRP3 and activates caspase-1 p20 expression and caspase-1 activity	Tan et al. (2019)
	ApoE−/− mice	Mice fed with diet containing 0.3% TMAO have higher TG, TC, LDL-C and increased total plaque areas in the aortas	Senthong et al. (2016)
	2,235 patients with stable CVD	Higher plasma TMAO levels are associated with a 4-fold increased mortality risk	Tan et al. (2019)
	211 patients with ST-segment-elevation myocardial infarction	Plasma TMAO levels are significantly higher in patients with plaque rupture than in those with plaque erosion	Liu et al. (2018)
	90 patients with CVD	high TMAO group exhibited a thinner fibrous cap thickness	Bordoni et al. (2020)
	394 CVD patients	No differences between CVD patients and control subjects in plasma TMAO levels	Zhu et al. (2020)
SCFAs	ApoE−/− mice	Aortic atherosclerotic plagues with a more durable fibrous cap are decreased by 50% with a diet containing 1% butyrate	Mathew et al. (2014)
	ApoE−/− mice	Intestinal administration of butyrate reduces endotoxaemia and atherosclerosis development	Bartolomaeus et al. (2019)
	ApoE−/− mice	Aortic atherosclerotic lesion area is decreased with propionate in the drinking water	Koh et al. (2016)
	ApoE−/− mice	HFD-induced AS are inhibited and ABCA1 is significantly induced by butyrate	Kuipers et al. (2014)
BAs	FXR−/− mice	Weight gain and hepatic steatosis in an FXR-dependent manner	Lambert et al. (2003)
	FXR−/− mice	Relate to impaired HDL metabolism and reverse cholesterol transport	Miyazaki-Anzai et al. (2014)
	ApoE−/− mice and LDLR−/− mice	Reduced atherosclerotic plaque formation and decreased expression of pro-inflammatory cytokines and chemokines in the aortas through the inactivation of NF-κB	Hanniman et al. (2005)
	ApoE−/− LDLR−/− mice	Loss of FXR function is associated with decreased survival, increased severity of defects in lipid metabolism, and more extensive aortic plaque formation	He et al. (2006)
	Rat	inhibition of ERK activation	Leuti et al. (2020)

TMAO, Trimethylamine N-oxide; CVD, cardiovascular disease; ApoE−/−, apolipoprotein E-deficient; AS, atherosclerosis; IL, interleukin; TNF-α, tumor necrosis factor-α; LDLR, low density lipoprotein receptor; MAPK, mitogen-activated protein kinase; ERK, extracellular signal-related kinase; NF-κB, nuclear factor-kappa B; EPCs, endothelial progenitor cells; FMD, flow-mediated vasodilation; hsCRP, high sensitivity C reactive protein; TG, triglyceride; TC, total cholesterol; LDL-C, low-density lipoprotein cholesterol; HFD, high fat diet; ABCA1, ATP-binding cassette sub-family A member 1; SCFAs, short-chain fatty acids; FXR−/−, farnesoid X receptor-deficient; HDL, high density lipoprotein; BAs, bile acids.

### Trimethylamine N-oxide

GM, especially those belonging to the Phylum Fusobacterium, *genus Clostridia* and Phylum Escherichia, *genus Escherichia coli*, process dietary phosphatidylcholine and l-carnitine into Trimethylamine (TMA; Brown and Hazen, 2015). Through the portal circulation, TMA is transported to the liver, where it is converted to TMAO by hepatic flavin monooxygenase 3 (FMO3). The suppression of the FMO3 gene has been shown in certain studies to greatly lower the formation of TMAO (Rath et al., 2017; Luo et al., 2022). Plasma levels of TMAO are very variable both within and across individuals, making it difficult to compare research (Papandreou et al., 2020).

TMAO is different from traditional CVD risk factors in that it is produced by gut microbes. More experiments in recent years have discovered that TMAO may become a whole new marker for predicting future CVD risk, with an emphasis on the proper serum levels to diagnose CVDs (Zheng et al., 2019; Tang et al., 2021). Several innovative techniques to inhibit TMAO production and prevent AS have been discovered in recent years (Iglesias-Carres et al., 2021; Li et al., 2021). Researchers reveal in one experiment that a high-choline diet offered to mice increases intraplaque bleeding rather than changing atherosclerotic load or plaque composition, which is a very new result (Lindskog Jonsson et al., 2018). As part of immune defense, TMAO activates the heat shock protein 60, which has been demonstrated to initiate AS and to participate in foam cell formation *via* Toll like receptors, which are also activated by SR-A1 and CD36 in macrophages after TMAO stimulation (Febbraio et al., 2000; Wick et al., 2004, 2014; Collot-Teixeira et al., 2007). Apart from that, TMAO can activate NLRP3, which can lead to IL-1β, IL-6 and tumor necrosis factor (TNF)-α production, triggering inflammation and endothelial injury that eventually leads to AS (Wu et al., 2020). TMAO also promotes the AS development by enhancing the production of IL-1β, IL-6, TNF-α and CRP through the activation of mitogen-activated protein kinase (MAPK) and NF-kappa B (NF-κB) signaling (Seldin et al., 2016; Chen et al., 2017; Chou et al., 2019). Additionally, TMAO could reduce reverse cholesterol transport by activating the nuclear receptor Farnesoid X receptor (FXR), which in turn might reduce bile acid synthesis in the liver with the effect of accelerating AS development (Ding et al., 2018; Tan et al., 2019).

In such cases, lowering TMAO to lower the AS incidence using certain effective, safe techniques might have a big impact on public health. However, the majority of *in vivo* investigations are undertaken in mouse models, with only a handful of people. According to Senthong et al. (2016) and Tan et al. (2019) plasma TMAO levels are closely related to coronary atherosclerotic burden in patients with ST-segment elevation myocardial infarction (STEMI) and stable coronary artery disease (CAD). Liu et al. (2018) previously demonstrate that non-culprit plaques in CAD patients with greater TMAO levels have more susceptible features, including a thinner fibrous cap, a higher presence of thin-cap fibroatheroma and microvessels. However, A comparison of plasma TMAO levels in CAD patients and control subjects reveal no significant differences in another trial (Bordoni et al., 2020).

TMAO plays a role in the complex pathological processes of atherosclerotic lesion formation. The preliminary findings suggest that TMAO might serve as a potential target for the prevention and treatment of AS, which is conceptually novel, compared with existing treatments. The regulating of TMAO production and associated GM may be an effective strategy against AS (Zhu et al., 2020).

### Short-chain fatty acids

Certain gut microbes produce SCFAs (such as acetate, proprionate, and butyrate) by fermenting otherwise indigestible dietary fibers. It has been shown that fecal and plasma levels of SCFA are related to the presence of SCFA-producing microbiota in the gut and the consumption of dietary fibers (Calderon-Perez et al., 2020; Deehan et al., 2020; Li et al., 2020; Verhaar et al., 2020). SCFAs are absorbed and delivered through the portal circulatory system to control immunological responses and a variety of metabolic activities in the host (Wenzel et al., 2020; Yang et al., 2020). SCFAs have emerged as beneficial biological microbial products with well-known anti-atherogenic properties, such as lowering systemic inflammation, increasing endothelial cell function, and maintaining intestinal barrier integrity (Lin and Zhang, 2017).

According to a study, aortic atherosclerotic plagues are decreased by 50% when apolipoprotein E (ApoE)^−/−^ mice had a diet containing 1% butyrate for 10 weeks, with lower macrophage infiltration and greater collagen deposition, implying a more durable fibrous cap. These events are largely linked to a decrease in CD36 in macrophages and endothelial cells, as well as a decrease in pro-inflammatory cytokines and NF-κB activation (Aguilar et al., 2016). Butyrate also can prevent inflammation development by effecting proliferation of VSMCs (Ranganna et al., 2003; Mathew et al., 2014). Atherosclerotic GF ApoE^−/−^ mice were inoculated with a specified population of eight bacteria. The size of atherosclerotic plaques is decreased by 30% using that axis without changing cholesterol or TMAO levels, possibly due to SCFAs’ capacity to keep the gut healthy (Bultman, 2018; Kasahara et al., 2018). Furthermore, Bartolomaeus et al. (2019) discover that improving vascular inflammation and atherosclerotic lesion load in ApoE^−/−^mice, as well as lowering blood pressure, all of which are classic plaque rupture risk factors. In addition to mitigating CVD risks, they have also been useful in mitigating diabetes, obesity, hypertension (Koh et al., 2016), etc. SCFAs improves gut barrier integrity, vascular dysfunction, hypertrophy fibrosis, and cardiac ventricular arrhythmias in ApoE^−/−^ mice (Du et al., 2020).

In GM-AS disease axis research, SCFA butyrate is therefore believed to act antagonistically to its counterpart secondary metabolite-TMAO. It might be a novel strategy—supplementing SCFAs to prevent AS and stabilize plaque in the event of a cardiovascular event. However, because few human studies have been completed, additional research into the role of SCFAs is still needed.

### Bile acids

Another group of GM-produced metabolites involved in various metabolic disorders is BAs, which are stored in the gallbladder and released into the intestine to aid dietary lipid absorption and dietary fat-soluble vitamin absorption (Kuipers et al., 2014). The GM-derived enzymes usually transform primary BAs (such as deoxycholic acid, lithocholic acid hyodeoxycholic acid, and ursodeoxycholic acid) into secondary BAs. The enterohepatic system of GF mice exhibit higher levels of primary BAs, but no secondary BAs (Chiang, 2013). High fat diet (HFD)-induced gut microbiome changes are inhibited when liver BA biosynthesis is suppressed. This illustrates a metabolic link between the liver, the microbiome of the gut, and the BAs (Zheng et al., 2017). Consequently, there exists a bidirectional relationship between GM and BAs metabolism (Yan et al., 2021).

Through the BAs receptor (also known as FXR), the GM can affect lipid and cholesterol metabolism. It is vital for the absorption of dietary lipids and fat-soluble vitamins that bile acids are present (Parseus et al., 2017). Upon feeding mice a chow diet, FXR-deficient mice develop hypercholesterolemia, related to impaired high density lipoprotein (HDL) metabolism and reverse cholesterol transport (Lambert et al., 2003). Studies using FXR agonists in mice prone to AS have also demonstrated that this receptor may contribute to AS prevention, but this effect may be weight-dependent (Miyazaki-Anzai et al., 2014). Moreover, double-knockout mice for ApoE and FXR have a deteriorated plasma lipid profile and more atherosclerotic lesions compared to ApoE^−/−^ mice (Hanniman et al., 2005). Since FXR is expressed by aortic smooth muscle cells and endothelial cells, this leads to aortic smooth muscle cell injury (He et al., 2006; Zhang et al., 2008). Several FXR-expressing tissues may be involved in atherogenesis, and the role of microbial regulation of BAs is not fully understood. Animal studies account for the majority of current data, while human data is sparse.

## GM and inflammation in AS

Several diseases are characterized by inflammation, such as AS, cancer, asthma, autoimmune disorders and neurodegenerative disorders (Leuti et al., 2020). Pathogens are prevented by the epithelium of the gut, the first barrier of the host. In the presence of an impaired intestinal epithelial barrier, invasion of pathogen-associated molecular patterns (PAMPs) elicits an immune response, leading to systemic and tissue-specific inflammation (Desai et al., 2016). Dysbiosis of the gut has been identified as a risk factor for chronic inflammation, consequently reducing gut barrier integrity (Barcik et al., 2020).

Lipopolysaccharide (LPS) and peptidoglycan are microbial components known to contribute to CVD (Roberts and Buford, 2021). In 1999, plasma endotoxins are measured in the clinic to establish the link between LPS and CVD (Wiedermann et al., 1999). Gram-negative bacteria produce LPS as a component of their cell walls, which has been extensively studied in relation to CVD risk. Numbers of subsequent studies conducted by different groups of researchers have gradually confirmed the relationship (Battson et al., 2018; Asada et al., 2019; Yoshida et al., 2020). A significant increase in CVD is associated with the transportation of LPS derived from microbiomes. The LPS derived from gut dysbiosis may modulate Toll-like receptors (TLRs) and their downstream targets, such as myeloid differentiation factor88 (Myd88) and NF-κB, resulting in increased expression of pro-inflammatory cytokines and an increased risk of CVD (Guzzo et al., 2012; Zhao et al., 2021; Lai et al., 2022). Low-grade chronic inflammation triggered by LPS may also lead metabolic endotoxemia (ME), which is commonly observed in CVD patients. Increased intestinal permeability is associated with ME (Moludi et al., 2020).

Furthermore, another bacterial PAMPs—peptidoglycan (PG) has been linked to an increased risk of CVD *via* compromising the intestinal epithelial barrier (Han et al., 2021). PG is a small component from the cell wall of Gram-negative bacteria, whereas it is a large component of Gram-positive bacteria. Scientists discover that patients with AS have an amplification of genes that encoded PG production through metagenomic sequencing (Karlsson et al., 2012; Jin et al., 2021). Undoubtedly, atherosclerotic arteries are shown to include pro-inflammatory bacterial PG, which is linked to susceptible plaques (Laman et al., 2002).

Other PAMPs, such as CpG-oligodeoxynucleotides, flagellin, lipopeptides and others, can stimulate inflammatory processes by interacting with host pattern recognition receptors (Frostegard, 2022). All of the data points to functional alterations in the GM as a possible contributor to the development of AS risk. As shown in the Figure 1.

## Natural products regulate GM and inflammation in AS

Until recently, non-invasive therapeutic interventions for AS mostly concentrate on pharmaceutically reducing risk variables, such like arterial hypertension and hypercholesterolemia following healthy lifestyle choices. Inflammation, on the other hand, provides a series of pathways that connect established risk variables to altered arterial wall cell behavior and leukocyte recruitment. These pathways contribute to the progression of disease and its effects. Natural medicines have been the subjects of much research, and their clinical application as an appropriate and supplementary treatment is growing at an increasing pace across countries. Natural products formed from natural remedies are substances with a well-known chemical diversity and bioavailability. These items are still being used as a model for creating new pharmacological medications to prevent ailments like CVD (Zhou et al., 2020). Natural products have been proved to improve inflammation, foam cell production, lipid oxidation and abnormal platelet function (Hong et al., 2019).

The GM’s composition and activity can affect intestinal permeability, digestion, metabolism and immunological responses. Alterations in GM equilibrium generate a pro-inflammatory status, which contributes to the beginning of a variety of illnesses varying from gastrointestinal and metabolic disorders to immunological disorders (Gomaa, 2020). In recent years, the GM sector has shifted its attention to natural products and medical research, as well as attempts to recognize natural products’ pharmacological activities. As shown in the Figure 2.

### Berberine

BBR is a major bioactive component of Coptis chinensis and other Berberis plants, which compound with numerous pharmacological properties. In addition to improving cardiovascular hemodynamics, BBR inhibits ischemic arrhythmia, slows down AS development, and reduces hypertension (Cicero and Baggioni, 2016; Song et al., 2020). The mechanisms of BBR include platelet activation inhibited, endothelial function normalized, macrophage-derived foam cell generation suppressed, inflammation and inflammasome activation inhibited and so on (Feng et al., 2019). It also has antibacterial properties, preventing bacterial reproduction (Zhang et al., 2021).

BBR increases gut microbial community, decreases intestinal pH and exogenous antigen deposition, raises LPS-binding protein and leptin levels, decreases serum adiponectin, has lipid-lowering and hypoglycemic benefits, boosts antibiotic culture and SCFAs synthesis (Han et al., 2011; Zhang et al., 2012). BBR boosts the synthesis of SCFAs in these phyla: Roseburia, Blautia and Alistipes (Wu et al., 2020). Cohousing BBR-treated mice with HFD-fed animals inhibits atherosclerotic progression in a comparable way to non-cohoused BBR-treated mice (Shi et al., 2018). In addition to this, Wu et al. further argue that different doses of BBR demonstrate different sensitivity towards GM composition. High doses of BBR display profound GM metabolic activity through the production of SCFAs. This activity is linked to BBR-induced GM compositional shift (Zhu et al., 2018).

By restoring intestinal SCFAs levels, reducing serum LPS levels, reducing intestinal inflammation and repairing intestinal barrier integrity, BBR treatments proved effective (Zhang et al., 2019). The BBR also reverses structural and compositional changes in the GM and influences gut microbe-dependent metabolic pathways (Yang et al., 2021). These results suggest that BBR interacts with GMs in order to play a regulatory role in dysmetabolism and AS.

### Anthocyanin

Anthocyanin is flavonoid polyphenols with antioxidant properties that have been associated with reduced CVD risk (Aboonabi and Singh, 2015; Garcia and Blesso, 2021). Isolated anthocyanin successfully modifies the “pro-atherogenic” GM communities by raising percentages of five *genera* (*Bifidobacterium, Lactobacillus, Roseburia, Akkermansia and Parabacteroides*; Lavefve et al., 2020; Si et al., 2021).

In addition to controlling GM, Anthocyanin enhances vascular function by increasing nitric oxide (NO) production, lowering oxidative stress, and decreasing aortic inflammation. The aortas from these animals have much less leukocyte infiltration, less plaque development and lower quantities of blood inflammatory cytokines than those in the control mice (Joo et al., 2018; Festa et al., 2021). The NF-κB inflammatory pathway, hepatic lipid metabolism and cell redox state are all under the influence of Anthocyanin at the same time (Mena et al., 2014).

In cultured macrophages and endothelial cells, Anthocyanins may also change the mRNA levels of genes associated with AS. According to a nutrigenomic research, Anthocyanins alters the expressions of 1,261 genes in the aorta, which are linked to the development and prevention of AS (Mauray et al., 2012). Protocatechuic acid is a kind of Anthocyanin metabolites that partially inhibits AS by activating the miRNA-10b-ABCA1/ABCG1-cholesterol efflux signaling pathway (Wang et al., 2012).

As a result, Anthocyanin-mediated activities significantly alleviates AS, however it is unclear how these effects would translate in “real world” with GM confounders.

### Polymethoxyflavones

PMFs, which are abundant in old citrus peels, have a wide range of biochemical functions, including antioxidant and anti-inflammatory properties (Lai et al., 2015). Aside from a well broad spectrum of their anti-AS properties, the current wave of microbial investigation has motivated scientists to explore their influence on GM balance (Yen et al., 2011).

PMFs change the GM structure to a healthy phenotype by boosting *Akkermansia, Lactobacillus, Bacteroides, and Bifidobacterium genera*. They increase butyrate-producing bacteria and reduces TMA-producing microorganisms, respectively. PMFs also suppress hepatic FMO3, which reduce the conversion of L-carnitine to TMAO. Apart from inhibiting the TMAO synthesis route, PMFs can also decrease the NF-κB and MAPK signaling pathways, preventing vascular inflammation (Yang et al., 2019). By lowering markers of inflammation (VCAM-1, TNF and E-selectin) and TMAO, PMFs reliably reduce L-carnitine-induced vascular inflammation. Therapy with PMFs further reduce the development of macrophage foam cells (Whitman et al., 2005; Chen et al., 2019). By using high performance liquid chromatography electrospray ionization tandem mass spectrometry, a total of 21 PMF metabolites that are discovered (Chen et al., 2020).

### Quercetin

Quercetin is a flavonoid that may be found in a variety of foods (onions, tea, apples, etc.) and reduces platelet aggregation and thrombus development in human participants (Hubbard et al., 2004). Similarly, it benefits cholesterol metabolism, intestinal inflammation, atherosclerotic lesions and sizes of plaques, and promote gut health (Nie et al., 2019; Uyanga et al., 2021).

Quercetin has been found to have concentrated action in GM regulation, which is useful in helping mice recover their GM after receiving antibiotics and may serve as a prebiotic in the fight against gut dysbiosis (Shi et al., 2020; Zhao et al., 2021). It’s worth noting that 32 metabolic markers are discovered to drive Quercetin’s effects. The major BA production route, specifically 3, 7, 12, and 26-tetrahydroxy-5-cholestane, is one of them (C_27_H_48_O_4_). This suggests Quercetin may increase the GM metabolite BA, correlated with circulating BAs and liver lipid and BA metabolism genes (Juarez-Fernandez et al., 2021). BAs, as aforementioned, play an important role in maintaining human metabolic processes such adipose tissue browning, insulin sensitivity and intestinal barrier integrity (Chiang and Ferrell, 2020). Additionally, intestinal Cu/Zn-superoxide dismutase, glutathione peroxidase and nutritional transporters such glucose transporter 2, peptide transporter 1 and fatty acid synthase genes have significantly higher levels of mRNA expression in the groups who received quercetin supplements (Abdel-Latif et al., 2021). With the consequent suppression of the inflammasome sensitivity and stimulation of the reticulum stress pathway, quercetin restores GM imbalance and associates with endotoxemia-mediated TLR-4 pathway stimulation (Porras et al., 2017). Quercetin and Resveratrol have positive impacts in improving the dysbiosis of the GM and obesity brought on by the HFD. They attenuate blood lipids, serum IL-6 and TNF-α and monocyte chemotactic protein (MCP)-1. Significantly limit the relative abundance of Desulfovibrionaceae, Acidaminococcaceae, Coriobacteriaceae, Bilophila, Lachnospiraceae family and result in a decrease in the F/B ratio (Zhao et al., 2017).

### Ferulic acid

FA is a well-known phenolic compound that has anti-inflammatory and antioxidant properties. It helps the metabolism of lipids, diabetes and thrombosis in addition to having cardioprotective and antihypertensive effects (Neto-Neves et al., 2021). A study shows that FA can lower blood cholesterol, triglyceride and LDL levels as well as the ratio of F/B in GM composition (Ma et al., 2019). Additionally, FA lowers the expression of peroxisome proliferator-activated receptor (PPAR)-β and PPAR-γ and increases the expression of PPAR-α, a gene linked to fat metabolism. The fatty acid production and metabolism benefits from these alterations in gene expression, which also reduces fat accumulation (Yin et al., 2022).

In mice, FA can modulate the GM and inflammation and drastically reduce AS. The GM and fecal metabolites undergo noticeable structural alterations due to FA. In contrast to enhancing the Lachoiraceaea family, FA can reduce the levels of IL-1β, IL-2, IL-6, TNF-α and relative proportions of the Prevotellaceae family (Hu et al., 2022).

### Pomegranate juice

PJ is high in polyphenols and is renowned for its anti-AS and anti-inflammation characteristics (Danesi and Ferguson, 2017). PJ results in a significant reduction of the atherosclerotic lesion size and intima media thickness, a 20% reduction in the absorption of ox-LDL and native LDL by peritoneal macrophages (Aviram et al., 2000; Basu and Penugonda, 2009), a 27% rise in HDL content and a 12 to 18% reduction in the risk of CVD (Michicotl-Meneses et al., 2021).

In RAW264.7 macrophages, PJ stimulates cholesterol efflux from foam cells and inhibits the pathophysiology of initial atherogenesis (Zhao et al., 2016). The expression of vascular inflammation indicators, arterial endothelial nitric oxide synthase (eNOS), and levels of inflammatory cytokines [TNF-α, plasminogen activator inhibitor-1 (PAI-1), IL-17A, IL-6, IL-1β, and MCP-1] all show a considerable decline (de Nigris et al., 2007; Michicotl-Meneses et al., 2021). A PJ supplementation reduces oxidative stress in macrophages and cholesterol flux in macrophages, even also attenuates AS in mice (Kaplan et al., 2001). A dose-dependent impact of PJ and its primary polyphenols decreases TNF-α and IL-6 secretion significantly. A considerable increase in the spontaneous release of IL-10 revealed that PJ dose-dependently influences macrophages toward an anti-inflammatory M2 phenotype (Aharoni et al., 2015).

After using PJ supplements, the levels of the phenolic metabolites 3-phenylpropionic acid, catechol, hydroxytyrosol and urolithin A in the feces significantly increase (Mosele et al., 2015). PJ is reported to reverse acrolein-induced GM dysbiosis by rebalancing the F/B ratio and lowering the prevalence of Ruminococcaceae and Lachnospiraceae at the family level. *In vivo* and *in vitro* atherosclerotic models, it also preventes acrolein-induced lipid buildup, peroxidation and oxidative stress by decreasing critical lipid biosynthesis pathways (Rom et al., 2017). Furthermore, PJ with chitinglucan reduces the relative abundance of phylum Bacteroidetes, *genus Alistipes* and Phylum Firmicutes, *genus Lactobacillus* through altering their genes, also reduces hepatic lipids, inflammation and endothelial dysfunction through activating eNOS (Neyrinck et al., 2019).

### Resveratrol

RSV is a stilbenoid phenol that may be discovered in grapes, blueberries and medicinal herbs, which can reduce intestinal permeability and inflammation and enhance gut barrier (Cai et al., 2020). It is also effective anti TMAO-induced AS in a GM-dependent way and increase the abundance of *genera Lactobacillus* and *Bifidobacterium* (Chen et al., 2016).

RSV has been found by researchers to have anti-inflammatory effects and to have the ability to prevent the generation of inflammatory cytokines. This anti-inflammatory impact may be linked to the modulatory effect of microRNAs transcription with either an anti-inflammatory or a pro-inflammatory role (Dvorakova and Landa, 2017), which is predominantly mediated by the capacity to regulate NF-κβ and activator protein-1 (AP-1; Latruffe et al., 2015). In this setting, RSV has been shown to be able to reduce chronic low-grade inflammation, which is defined by the buildup of macrophages in the adipose tissue and excessive cytokine production. For instance, it reduces the release of TNF-α, IL-1β, IL-6 and IL-8 in murine adipocytes and human adipose tissue explants (Olholm et al., 2010; Yen et al., 2011). RSV may reduce inflammation linked to obesity in genetically (Gomez-Zorita et al., 2013). Even more, it can have an immediate anti-inflammatory impact in healthy persons (Ghanim et al., 2011).

RSV reduces the GM structural alterations and inflammatory markers by HFD (Man et al., 2020). *Genera Bacteroides, Blautia, Lachnoclostridium, Parabacteroides* and *Ruminiclostridium 9* are enriched in the gut of mice treated with RSV, which is a striking modification in the makeup of the microbiota (Wang et al., 2020). The anti-inflammation benefits of RSV are substantially dependent on GM, as demonstrated by a fecal microbiota transplantation experiment (Wang et al., 2020, 2022). RSV also successfully stops the growth of Coriobacteraceae and Desulfovibrionaceae, two family that have a strong relationship with body weight that are caused by the HFD (Campbell et al., 2019).

### Tea polyphenols

Given atherosclerotic circumstances, TPs are reported to exhibit a wide range of biological actions, which have antioxidants and anti-inflammatory effects (Bornhoeft et al., 2012).

TPs decrease plaque/lumen area and increase *Bifidobacterium* colony abundances in the intestines of HFD-fed ApoE^−/−^ mice. Furthermore, the rise in *Bifidobacterium* count is approximately equal to the plaque/lumen size. This means that TPs target *Bifidobacterium* specifically to reduce atherosclerotic plaque (Liao et al., 2016). TPs increase SCFAs synthesis in C57BL/6 J mice with human flora (Wang et al., 2018). TPs may decrease systemic LPS levels and inflammation brought on by obesity. Toll-like receptor 4 (TLR4), as well as CD14, MyD88 and other co-receptors, are downregulated in hepatic and adipose tissues in response to the drop in the level of LPS in the blood. This further prevents NF-κB to be activated. In mice fed with HFD, TPs dramatically reduce the blood levels of TNF-α, IL-1β and IL-6. By boosting intestinal tight junction proteins, TPs also preserve the integrity of the intestinal barrier and repair gut dysbiosis in obese mice (Li et al., 2020; Ye et al., 2022).

The performance of the barrier function, particularly intestinal inflammation and the integrity of the intestinal barrier, are improved by TPs. They also reduce the phylogenetic variation and F/B ratio associated with GM dysbiosis brought on by HFD. Other fundamental bacteria are discovered to benefit from TPs include *genera Akkermansia muciniphila, Alloprevotella, Bacteroides* and *Faecalibaculum* (Zhou et al., 2021). Another study finds that TPs increase the relative abundance of phylum Firmicutes while decreasing the relative number of *genera Bacteroidetes and genera Fusobacteria* by 16S rRNA gene sequencing. In addition, significant correlations exist between the weight loss caused by TPs and the relative amount of the *genera Acidaminococcus, Anaerobiospirillum, Anaerovibrio, Bacteroides, Blautia, Catenibactetium, Citrobacter, Clostridium, Collinsella* and *Escherichia* (Li et al., 2020).

## Conclusion

Natural products can reduce the pro-inflammation cytokines and inhibit inflammation-related signaling pathways in AS-related CVD by regulating the GM composition. The interplay of natural products with GM and inflammation gives unique insights into the gut-heart axis, expanding routes and concepts for using medicinal plants and naturally derived medications. Further research encompassing pharmacodynamics, pharmacokinetics, microbiomics, and metabolomics is urgently required to have a thorough understanding of this developing field in terms of creating innovative therapeutic methods for the prospective therapy of atherosclerotic CVD.

## Author contributions

LT wrote the first draft of the review. BD compiled the tables and figures, and all the revisions according to the reviewers. YW accepts overall responsibility for the final submitted version of this review. All authors contributed to the article and approved the submitted version.

## Funding

This study was supported by the National Natural Science Foundation to YW (grant number 82204849), Traditional Chinese Medicine Research Project of Shanghai Municipal Health Commission to YW (grant number 2022QN056), Clinical Technology Innovation Cultivation Program of Longhua Hospital Affiliated to Shanghai University of Traditional Chinese Medicine to YW (grant number PY2022008), and Regional medical Centre of Longhua Hospital Affiliated to Shanghai University of Traditional Chinese Medicine to YW (grant number ZYZK001-029).

## Conflict of interest

The authors declare that the research was conducted in the absence of any commercial or financial relationships that could be construed as a potential conflict of interest.

## Publisher’s note

All claims expressed in this article are solely those of the authors and do not necessarily represent those of their affiliated organizations, or those of the publisher, the editors and the reviewers. Any product that may be evaluated in this article, or claim that may be made by its manufacturer, is not guaranteed or endorsed by the publisher.

## Figures and Tables

**Table:** 

GM	Gut microbiota
AS	Atherosclerosis
CVD	Cardiovascular disease
ox-LDL	Oxidized low-density lipoprotein
LDL	Low density lipoprotein
VCAMs	Vascular cell adhesion molecules
VSMCs	Vascular smooth muscle cells
CANTOS	Canakinumab Anti-inflammatory Thrombosis Outcome Study
IL	Interleukin
COLCOT	Colchicine Cardiovascular Outcomes Trial
LoDoCo2	Low-Dose Colchicine 2 trial
ACS	Acute coronary syndromes
CRP	C-reactive protein
CIRT	Cardiovascular Inflammation Reduction trial
DNA	Deoxyribonucleic acid
SPF	Specific-pathogen-free
GF	Germ-free
LDLR^−/−^	LDL receptor-deficient
F/B	Firmicutes/Bacteroidetes
TMAO	Trimethylamine N-oxide
TMA	Trimethylamine
FMO3	Flavin monooxygenase 3
TNF	Tumor necrosis factor
MAPK	Mitogen-activated protein kinase
NF-κB	NF-kappa B
FXR	Farnesoid X receptor
STEMI	ST-segment elevation myocardial infarction
CAD	Coronary artery disease
SCFAs	Short-Chain Fatty Acids
ApoE	Apolipoprotein E
BAs	Bile acids
HFD	High fat diet
HDL	High density lipoprotein
PAMPs	Pathogen-associated patterns
LPS	Lipopolysaccharide
TLRs	Toll-like receptors
PG	Peptidoglycan
BBR	Berberine
NO	Nitric oxide
PMFs	Polymethoxyflavones
MCP	Monocyte chemotactic protein
FA	Ferulic acid
PJ	Pomegranate juice
eNOS	Endothelial nitric oxide synthase
PAI-1	Plasminogen activator inhibitor-1
RSV	Resveratrol
AP-1	Activator protein-1
TPs	Tea polyphenols
TLR4	Toll-like receptor 4.
